# Impact of cancer on short-term in-hospital mortality after primary acute myocardial infarction

**DOI:** 10.1136/openhrt-2021-001860

**Published:** 2021-11-22

**Authors:** Robert Zheng, Kenya Kusunose, Yuichiro Okushi, Yoshihiro Okayama, Michikazu Nakai, Yoko Sumita, Takayuki Ise, Koji Yamaguchi, Shusuke Yagi, Daiju Fukuda, Hirotsugu Yamada, Takeshi Soeki, Tetsuzo Wakatsuki, Masataka Sata

**Affiliations:** 1 Department of Cardiovascular Medicine, Tokushima University Hospital, Tokushima, Japan; 2 Center for Cerebral and Cardiovascular Disease Information, National Cerebral and Cardiovascular Center, Osaka, Japan; 3 Department of Community Medicine for Cardiology, Tokushima University Graduate School of Biomedical Sciences, Tokushima, Japan

**Keywords:** myocardial infarction, risk factors, acute coronary syndrome

## Abstract

**Background:**

Cardiovascular diseases are the second most common cause of mortality among cancer survivors, after death from cancer. We sought to assess the impact of cancer on the short-term outcomes of acute myocardial infarction (AMI), by analysing data obtained from a large-scale database.

**Methods:**

This study was based on the Diagnosis Procedure Combination database in the Japanese Registry of All Cardiac and Vascular Diseases and the Diagnosis Procedure Combination. We identified patients who were hospitalised for primary AMI between April 2012 and March 2017. Propensity Score (PS) was estimated with logistic regression model, with cancer as the dependent variable and 21 clinically relevant covariates. The main outcome was in-hospital mortality.

**Results:**

We split 1 52 208 patients into two groups with or without cancer. Patients with cancer tended to be older (cancer group 73±11 years vs non-cancer group 68±13 years) and had smaller body mass index (cancer group 22.8±3.6 vs non-cancer 23.9±4.3). More patients in the non-cancer group had hypertension or dyslipidaemia than their cancer group counterparts. The non-cancer group also had a higher rate of percutaneous coronary intervention (cancer 92.6% vs non-cancer 95.2%). Patients with cancer had a higher 30-day mortality (cancer 6.0% vs non-cancer 5.3%) and total mortality (cancer 8.1% vs non-cancer 6.1%) rate, but this was statistically insignificant after PS matching.

**Conclusion:**

Cancer did not significantly impact short-term in-hospital mortality rates after hospitalisation for primary AMI.

**Key questions**

**What is already known about this subject?**

- Some reports have identified cardiovascular diseases as being the second most common cause of mortality among cancer survivors, after death from cancer.

**What does this study add?**

- Cancer did not significantly impact short-term in-hospital mortality rates after hospitalisation for primary acute myocardial infarction (AMI).

**How might this impact on clinical practice?**

- It is of great importance to know the risk factors of deaths in AMI and cancers.

## Introduction

With advances in detection and treatment methods, survival rates and overall survival have greatly improved in patients with cancer. As a result, cardiovascular diseases (CVDs) have become more prevalent in patients with cancer and cancer survivors.^1 2^ Some reports have identified CVDs as being the second most common cause of mortality among cancer survivors, after death from cancer. Many factors are thought to be attributed to this trend, such as the presence of common risk factors, inadequate management of these factors, attenuation of atherosclerosis arising from chemotherapy, radiation or the malignancy itself.^3–18^

Among the multiple CVDs observed in patients with cancer, ischaemic heart disease is relatively easy to prevent with proper intervention. However, with the large variety of malignancies and an even larger variety of treatments, patients with cancer are an extremely heterogenous group, with each individual cancer type potentially having differing risks and outcomes. In this study, we sought to assess the impact of cancer on the short-term outcomes of acute myocardial infarction (AMI), by analysing data obtained from a nationwide insurance claims database based on electronic health records in Japan.

## Methods

### Study population

Data from the Japanese Registry of All Cardiac and Vascular Diseases and the Diagnosis Procedure Combination (JROAD-DPC) database was used for this study. JROAD-DPC is a nationwide registry, with information of admission and discharge for CVDs, clinical examinations and treatment status, patient status and hospital overview. JROAD-DPC database integrates the information composed by JROAD-DPC data, with analysis data sets covering 5.1 million hospitalisations from 1022 facilities, between April 2012 and March 2017.^19^ The identification of AMI and cancer type was based on the International Classification of Diseases (ICD)-10 diagnosis codes related to AMI (I210, I211, I212, I213, I214, I219) and cancers of oesophagus (C15), stomach (C16), colon (C18-20), liver (C22), biliary tract (C23, 24), pancreas (C25), lung (C34), breast (C50), cervix (C53), uterine (C54), ovary (C56), prostate (C61), kidney and urinary tract (C64–66, 68), bladder (C67) and leukaemia (C91–95) based on our previous paper.^20^ Hospitalisation for AMI would be identified when AMI was registered as the main diagnosis, admission-precipitating diagnosis or being the most resource-consuming diagnosis. The most resource-consuming diagnosis is defined as the diagnosis for which the most medical resources are used, such as examinations, medications and treatments. Data regarding patient age and sex, main diagnosis, documented comorbidity at admission, length of hospitalisation and treatment content were extracted from the database.

**Figure 1:**
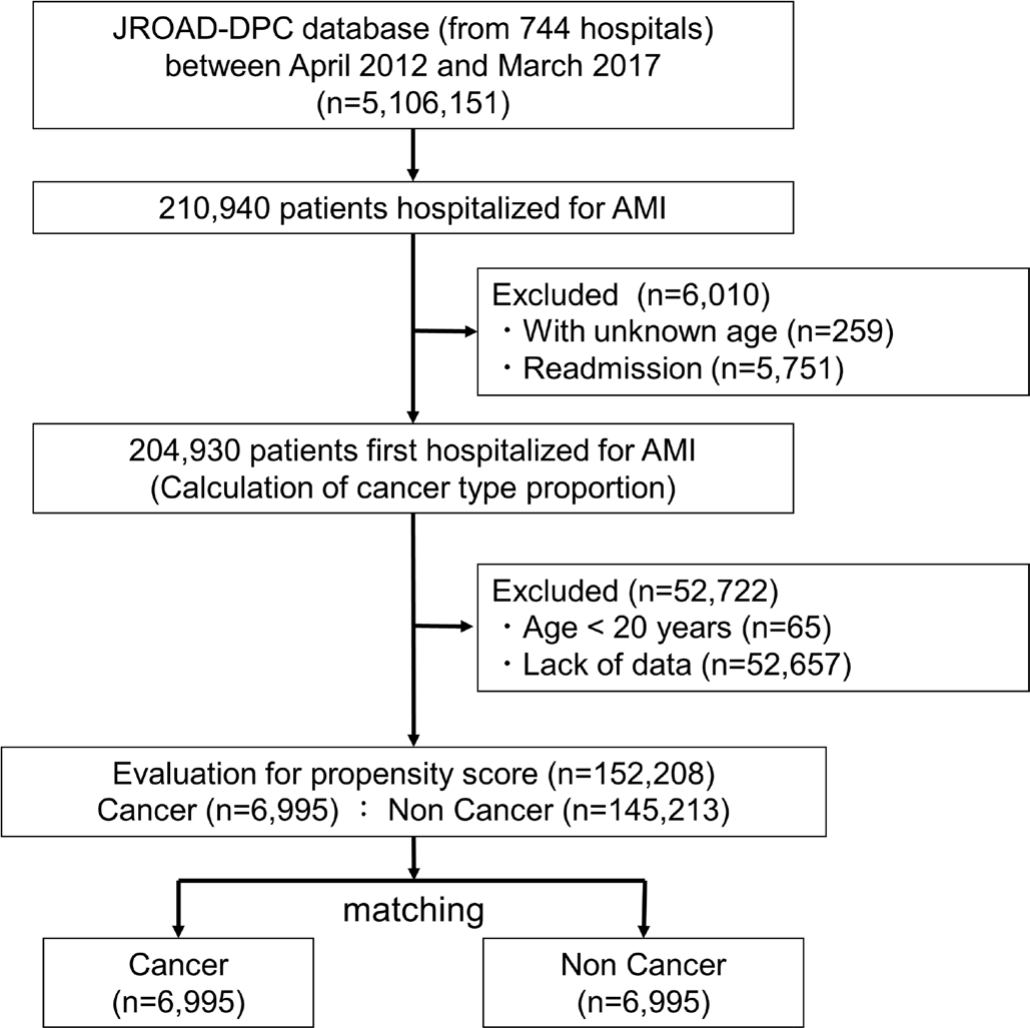
Flowchart of this study. AMI, acute myocardial infarction; JROAD-DPC, Japanese Registry of All Cardiac and Vascular Diseases and the Diagnosis Procedure Combination.

Out of a total of 5 106 151 hospitalisations extracted from the database, 2 10 940 patients were hospitalised for AMI. After exclusion of readmissions, 2 04 930 patients remained for calculation of cancer type proportion. A further 52 722 patients were excluded due to young age (<20 years) or incomplete data. The remaining 1 52 208 patients were split into a group with cancer (6995 patients) and those without cancer (1 45 213 patients) and were analysed with propensity matching (figure 1).

### Clinical outcomes

The main outcome was in-hospital mortality (total number of deaths during hospitalisation and death at 14 and 30 days after admission). Patients were censored on discharge and were not followed beyond that point.

### Sample matching

Propensity Score (PS) matching was used to reduce confounding effects related to differences in patient background. PS was estimated with a logistic regression model, with the following 21 clinically relevant covariates: age, sex, body mass index (BMI), smoking, Killip class, comorbidities (hypertension (HT), diabetes mellitus (DM), dyslipidaemia (DL), hyperuricaemia, stroke, peripheral vascular disease, renal disease, liver failure, chronic obstructive pulmonary disease, dementia) and treatment (percutaneous coronary intervention (PCI), coronary artery bypass graft, catecholamine use, intra-aortic balloon pumping, percutaneous cardiopulmonary support, chemotherapy). These covariates were chosen for their potential association as risk factors of AMI and in-hospital mortality in general. Matching was performed with greedy-matching algorithm (ratio=1:1 without replacement), with a calliper of width 0.2 SD of the logistic of the estimated PS. Absolute value of standardised differences less than 10% was considered to be statistically insignificant.

### Statistical analysis

The Shapiro-Wilk test was used to assess the normal distribution of continuous data. Continuous variables were expressed as mean±SD for parameters with normal distribution, as median (IQR) for parameters with skewed distribution and categorical variables as proportion (%). After PS matching, 6995 patients each in the cancer and non-cancer groups were included in the final analysis. Concordance Index was 0.667 and the consistency of PS densities was matched after PS matching. The balance of each covariate before and after the matching between the two groups was evaluated by standardised differences. Absolute value of standardised differences less than 10% was considered as a relatively small imbalance. We estimated the OR with cancer for in-hospital mortality (total, within 14 days and 30 days) major adverse cardiovascular events and major bleeding by matched logistic regression analysis adjusted for hospitalisation days. We also analysed subgroups by type of cancer in the PS-matched cohort. The OR for each type of cancer was calculated using matched non-cancer patients as controls. All statistical tests were two sided and p values less than 0.05 were considered statistically significant. Statistical analysis was performed using SAS V.9.4 and JMP V.14.0 (SAS Institute).

## Results

### Patient characteristics

**Figure 2:**
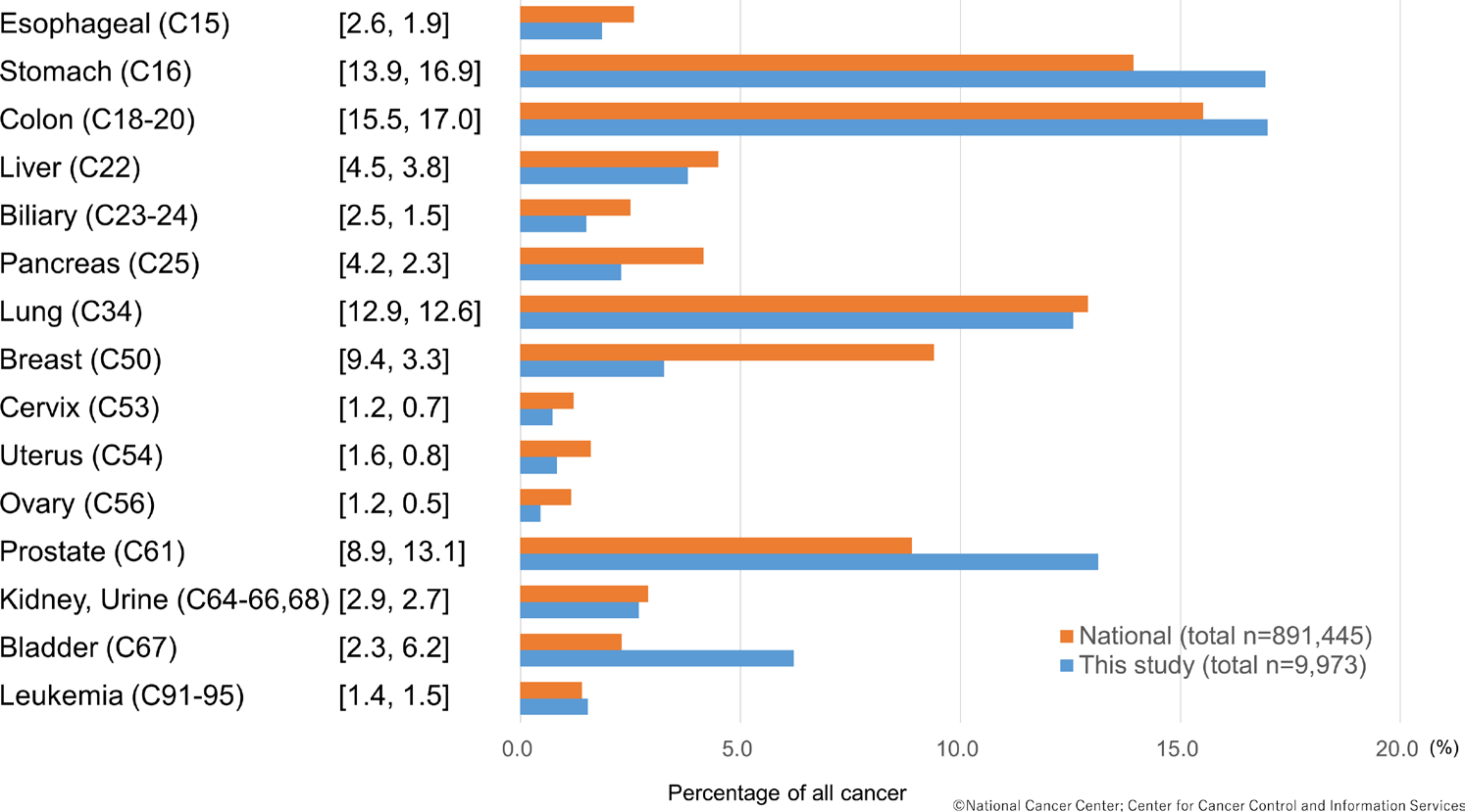
Proportion of cancer type of first hospitalised patients with acute myocardial infarction and comparison with national statistics (each cancer proportion of national statistics (%) and this study (%)).

The clinical characteristics of the cancer group and non-cancer group are shown in table 1. Patients with cancer tended to be older (cancer group 73±11 years vs non-cancer group 68±13 years) and had smaller BMI (cancer group 22.8±3.6 vs non-cancer 23.9±4.3). More patients in the non-cancer group had HT or DL than their cancer group counterparts. The non-cancer group also had a higher rate of PCI (cancer 92.6% vs non-cancer 95.2%). All the other clinical characteristics, including Killip class, showed no statistical difference between the two groups. The proportion of cancer types after the initial exclusion is shown in figure 2. Colon (17.0%), stomach (16.9%) and prostate (13.1%) cancers were the most numerous one. The cancer proportions found in our study were similar to the national trend of Japan, with slight differences in the percentage of prostate and breast cancer.

**Table 1:** Baseline characteristics before and after Propensity Score matching

	Non-matching				Matching		
	All	Cancer	Non-cancer	Std.diff (%)	Cancer	Non-cancer	Std.diff (%)
Number	(n=1 32 398)	(n=5852)	(n=1 26 546)		(n=5851)	(n=5851)	
Average age (years)	68±13	73±11	68±13	44.1	73±11	73±11	−1.3
Age (%)							
20–30	0.1	0.1	0.1	0.3	0.1	0.1	2.5
30–40	1.3	0.4	1.4	−10.9	0.4	0.5	−1.5
40–50	7.7	2.7	7.9	−23.4	2.7	3.2	−2.8
50–60	14.5	7.0	14.9	−25.5	7.0	7.6	−2.5
60–70	27.4	22.4	27.7	−12.1	22.4	22.8	−0.8
70–80	27.8	36.4	27.4	19.4	36.4	32.0	9.4
80–90	18.6	28.0	18.1	23.5	28.0	29.1	−2.5
>90	2.6	3.1	2.6	3.0	3.1	4.9	−9.3
Male (%)	76.1	76.5	76.1	1.0	76.6	76.5	0.2
BMI	23.9±4.3	22.8±3.6	23.9±4.3	−28.4	22.8±3.6	22.9±3.6	−1.4
Smoking	50.0	46.6	50.2	−7.3	46.6	46.9	−0.6
Killip							
1	51.4	48.2	50.2	−7.3	46.6	46.9	−1.3
2	27.9	28.8	27.8	2.2	28.8	29.0	−0.4
3	8.2	9.7	8.1	5.7	9.7	9.9	−0.6
4	12.6	13.2	12.5	2.0	13.2	12.3	2.9
Comorbidities (%)							
Hypertension	65.7	56.0	66.1	−20.8	56.0	56.9	−1.8
Diabetes mellitus	31.0	29.5	31.1	−3.5	29.5	29.6	−0.1
Dyslipidaemia	62.8	46.3	63.6	−35.3	46.3	46.8	−1.0
Hyperuricaemia	4.1	3.5	4.1	−3.2	3.5	3.3	1.1
Stroke	4.6	6.3	4.5	8.0	6.3	5.9	1.6
PVD	3.9	4.0	3.9	0.7	4.0	3.9	0.7
CHF	34.2	33.0	34.2	−2.7	33.0	32.2	1.7
CKD	4.5	5.8	4.5	6.1	5.8	5.7	0.5
Liver failure	<0.1	0.2	<0.1	4.4	0.2	0.1	0.8
COPD	2.4	4.0	2.3	9.8	4.0	4.0	0.2
Dementia	1.6	2.2	1.6	4.6	2.2	2.0	1.4
Treatment (%)							
PCI	95.1	92.6	95.2	−11.0	92.6	92.8	−1.0
DAPT usage	93.2	90.8	93.3	−9.3	90.8	91.4	−2.1
Stenting	87.8	82.5	88.0	−15.8	82.5	85.9	−9.6
CABG	2.7	2.4	2.7	−1.6	2.4	2.3	0.7
Catecholamine	41.9	45.9	41.7	8.6	45.9	45.1	1.6
IABP	17.1	18.4	17.1	3.6	18.4	18.0	1.1
PCPS	2.6	1.8	2.6	−5.2	1.8	1.8	0.2
Heart failure	0.3	7.5	<0.1	40.3	7.5	<0.1	40.3

Data are presented as percentage of patients or median (IQR). A standardised difference <10% suggests adequate balance.

BMI, body mass index; CABG, coronary artery bypass graft; CHF, congestive heart failure; CKD, chronic kidney disease; COPD, chronic obstructive pulmonary disease; DAPT, dual antiplatelet therapy; IABP, intra-aortic balloon pumping; PCI, percutaneous coronary intervention; PCPS, percutaneous cardiopulmonary system; PVD, peripheral vascular disease; Std.diff, standardisation difference.

### Outcomes

Patients with cancer had a higher 30-day mortality (cancer 6.0% vs non-cancer 5.3%) and total mortality (cancer 8.1% vs non-cancer 6.1%) rate (table 2), but this was statistically insignificant after PS matching. Bleeding events were significantly more frequent in the cancer group, with more patients requiring blood transfusion than the non-cancer group. The cancer group also had a higher rate of recurrent myocardial infarction (MI) (cancer 2.3% vs non-cancer 1.0%, p<0.001), while incidence rates of cerebral haemorrhages and infarctions showed no difference between the two groups. Further analysis of patients with recurrent MI (table 3) showed that patients with a cancer history were less likely to be selected for PCI (cancer 85.7% vs non-cancer 90.8%, p=0.041). Patients with cancer were less likely to underwent stenting (cancer 68.9% vs non-cancer 82.5%, p<0.001) or dual antiplatelet therapy (cancer 78.9% vs non-cancer 86.7%, p=0.007).

**Table 2:** Odds ratio of in-hospital mortality and MACE/bleeding incidence in patients before and after Propensity Score matching

	Non-matching				Matching			
	Cancer	Non-cancer	Adjusted OR* (95% CI)	P value	Cancer	Non-cancer	Adjusted OR* (95% CI)	P value
	(n=6995)	(n=1 45 213)			(n=6995)	(n=6995)		
In-hospital mortality								
Total (%)	567 (8.1)	8883 (6.1)	1.47 (1.35-1.61)	<0.001	567 (8.1)	606 (8.7)	0.94 (0.84 to 1.06)	0.345
7 days (%)	183 (2.6)	4823 (3.3)	1.49 (1.20-1.86)	<0.001	183 (2.6)	311 (4.5)	1.22 (0.93 to 1.61)	0.153
14 days (%)	282 (4.0)	6273 (4.3)	1.64 (1.41-1.91)	<0.001	282 (4.0)	413 (5.9)	1.16 (0.96 to 1.39)	0.133
30 days (%)	417 (6.0)	7729 (5.3)	1.69 (1.52-1.89)	<0.001	417 (6.0)	521 (7.5)	1.08 (0.94 to 1.24)	0.278
MACE								
Cerebral haemorrhage (%)	10 (0.1)	198 (0.1)	0.94 (0.50-1.79)	0.858	10 (0.1)	15 (0.2)	0.61 (0.27 to 1.37)	0.233
Cerebral infarction (%)	88 (1.5)	1377 (1.1)	1.12 (0.91-1.37)	0.292	105 (1.5)	88 (1.3)	1.07 (0.80 to 1.43)	0.644
Recurrent myocardial infarction (%)	161 (2.3)	1375 (1.0)	2.27 (1.92-2.68)	<0.001	161 (2.3)	86 (1.2)	1.80 (1.38 to 2.35)	<0.001
Major bleeding								
Gastrointestinal bleeding (%)	108 (1.5)	634 (0.4)	3.16 (2.57-3.89)	<0.001	108 (1.5)	29 (0.4)	3.40 (2.26-5.11)	<0.001
Blood transfusion (%)	698 (10.0)	4294 (3.0)	3.23 (2.96-3.52)	<0.001	698 (10.0)	274 (3.9)	2.01 (1.76-2.31)	<0.001

*Adjusted for hospitalisation days.

MACE, major adverse cadiovascular events; OR, odds ratio.

**Table 3:** Characteristics of patients with recurrent myocardial infarction after initial admission

N	Cancer	Non-cancer	P value
	161	1375	
Age (years)	74±10	71±13	<0.001
Male	83.9	69.1	<0.001
BMI	22.7±3.4	23.6±4.0	0.010
Smoking history	45.3	43.1	0.580
PCI selection	85.7	90.8	0.041
Stenting	68.9	82.5	<0.001
DAPT usage	78.9	86.7	0.007
Aspirin usage	88.2	92.2	0.079
Clopidogrel usage	52.2	59.1	0.092
Prasugrel usage	50.5	52.0	0.757

DAPT, dual antiplatelet therapy; PCI, percutaneous coronary intervention; PCI, percutaneous coronary intervention.

### Cancer types

**Figure 3:**
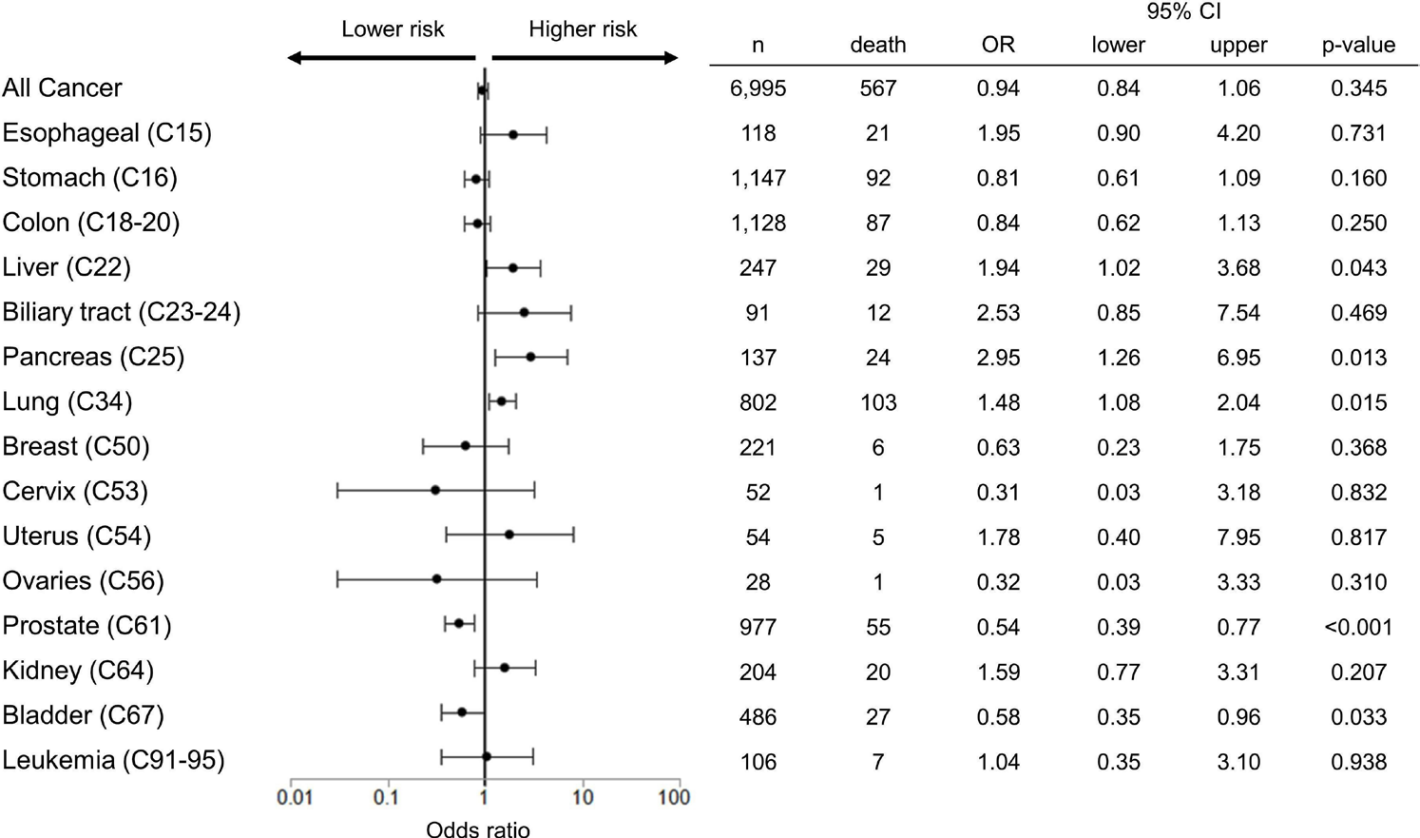
OR of in-hospital mortality in patients with each cancer compared with matched patients without cancer. Dots and lines mean OR and 95% CI, respectively.

The cancer types were generally spread out in multiple groups, though small in number. Regarding mortality rates in each cancer subtype, patients with pancreatic cancer (OR: 2.95, 95% CI: 1.26 to 6.95), liver cancer (OR: 1.94, 95% CI: 1.02 to 3.68) and lung cancer (OR: 1.48, 95% CI: 1.08 to 2.04) had higher mortality rates compared with non-cancer cohort (figure 3).

## Discussion

This analysis of data obtained from the J-ROAD DPC database revealed the following facts: (1) AMI patients with cancer were older and had lower BMI compared with their non-cancer counterparts, while having a lower prevalence of HT and DL, (2) patients with cancer had higher in-hospital mortality rates, but this difference was insignificant after PS matching and (3) patients with cancer had a higher rate of recurrent MI after initial hospitalisation.

### Cancer on mortality

Patients with cancer tended to be older (cancer group 73±11 years vs non-cancer group 68±13 years) and had smaller BMI (cancer group 22.8±3.6 vs non-cancer 23.9±4.3). Old age and lower BMI itself could be is generally related with higher overall mortality.^21^ It is worth noting that although there were many factors, older age, lower BMI and lower PCI rates, that could potentially affect overall mortality, cancer itself did not influence short-term in-hospital mortality after PS matching. PCI rates in the cancer group were not as low as past reports.^22–24^ Along with HT and DL, other conventional coronary risk factors were also less prevalent in patients with cancer, though not to an extent of statistical significance. There are several possibilities behind this trend. As mentioned in the previous sections, J-ROAD DPC is a database centred on cardiovascular centres, with these centres not necessarily having the capacity for cancer treatment. One possible reason for the increased prevalence of CVDs in cancer survivors is the fact that the management of occult atherosclerotic risk factors, such as HT, DL and DM, is not as sufficient as their non-cancer counterparts.^25–27^ In the case of this study, there is a possibility that some patients in the cancer group had previously undocumented comorbidities, not necessarily receiving proper treatment for such risk factors prior to hospitalisation. Another possible explanation for this trend is the existence of other atherosclerotic factors, such as prolonged inflammation from cancer, radiation therapy and chemotherapy. There is also a possibility that these factors contribute to accelerated atherosclerosis, even with the absence of contemporary coronary risk factors.

In this study, cancer did not show a significant increase in in-hospital mortality after PS matching. Being based on data obtained from a CVD -centred database compiled from data obtained from cardiovascular centres, cancer-oriented data, such as chemotherapy regimen, cancer staging and metastasis, are not included in the database. Data from this study at least indicated that the short-term outcome did not differ significantly with cancer.

### Cancer and recurrent MI

Analysis of major adverse cadiovascular events (MACE) after initial hospitalisation suggested that the cancer group had a higher incidence of recurrent MI. Secondary analysis of patients with recurrent MI showed a smaller rate of PCIs and antiplatelet usage in the cancer group. One possible explanation for this trend is higher bleeding risk in patients with cancer. Anaemia is more profound in patients with cancer, partly due to bleeding and myelosuppressive effects of chemoradiation therapies, further compounding the use of antiplatelet therapy. The hypercoagulative state in active cancers may also be another explanation.^28 29^

### Limitations

First of all, given the insurance claim-like nature of the database, detailed data, such as culprit lesion numbers/regions, stent types and dimensions and specific medication usage and doses, are not included in the original database, this would make it difficult to determine whether there were major differences in the management of AMI in both cancer/non-cancer groups. Particularly lacking information is detailed data on the use (and cessation) of antiplatelets/anticoagulants. The use of such medication may play a role in secondary bleeding/thrombotic events. Using a heterogenous group for analysis of the effects of cancer may diminish profound differences between cancer types, as some cancers may have higher bleeding risk than others. Second, J-ROAD DPC is a database based on data centred on CVDs, data specific to cancer, such as cancer stage (active or non-active), radiation treatment and chemotherapy regimens, were not included. We were unable to assess the differences among the types of AMI (type 1 or type 2, ST elevation or not). This database is large in scale but does not necessarily encompass the entire Japanese nation. Since this is a CVD database, medical centres solely purposed for cancer treatment and patients with cancer without documented CVDs may not be included, limiting the number and stages of patients with cancer. In this aspect, the result of this study may potentially underestimate the scope of which cancer affects the course of AMI. In our analysis, the number of patients for each cancer type is extremely small, with an even smaller number of mortalities. We attempted to analyse the mortality rates in each cancer type, but the statistical power of such an analysis weakens greatly with such minute numbers. Data are limited to in-hospital mortality rates, with each case censured on discharge. Therefore, status related to follow-up is not included in our study. Also, the accuracy of diagnosis is not complete in the JROAD-DPC database because of the low validity of these studies compared with planned prospective studies. However, the original JROAD-DPC dataset was validated.^30 31^ The registration of an ICD code for a cancer disease indicates that the cancer was associated with the treatment, which would at least suggest that it was a problematic condition of a history of or active cancer in the patient. However, as this database is based on data from cardiovascular centres, specific data on the state of each malignancy (active or past) were unavailable.

## Conclusions

Patients with cancer had a smaller proportion of HT and DL at admission for primary AMI. Cancer did not significantly impact short-term in-hospital mortality rates after hospitalisation for primary AMI.

## Data Availability

Data are available upon reasonable request. Individual anonymsed data supporting the analyses contained in the manuscript will be made available upon reasonable written request from researchers whose proposed use of the data for a specific purpose has been approved.
